# ATG4B Inhibitor UAMC-2526 Potentiates the Chemotherapeutic Effect of Gemcitabine in a Panc02 Mouse Model of Pancreatic Ductal Adenocarcinoma

**DOI:** 10.3389/fonc.2021.750259

**Published:** 2021-11-18

**Authors:** Farnaz Sedigheh Takhsha, Christel Vangestel, Muhammet Tanc, Sven De Bruycker, Maya Berg, Isabel Pintelon, Sigrid Stroobants, Guido R. Y. De Meyer, Pieter Van Der Veken, Wim Martinet

**Affiliations:** ^1^ Laboratory of Physiopharmacology, University of Antwerp, Antwerp, Belgium; ^2^ Molecular Imaging Center Antwerp (MICA), University of Antwerp, Antwerp, Belgium; ^3^ Department of Nuclear Medicine, University Hospital Antwerp, Edegem, Belgium; ^4^ Laboratory of Medicinal Chemistry, University of Antwerp, Antwerp, Belgium; ^5^ Department of Imaging Chemistry & Biology, King’s College London, London, United Kingdom; ^6^ Department of Science and Technology, AP University of Applied Sciences and Arts Antwerp, Antwerp, Belgium; ^7^ Infla-Med Centre of Excellence, University of Antwerp, Antwerp, Belgium; ^8^ Laboratory of Cell Biology and Histology, University of Antwerp, Antwerp, Belgium

**Keywords:** pancreatic ductal adenocarcinoma, autophagy, ATG4B, UAMC-2526, gemcitabine, proliferation, Panc02

## Abstract

Resistance against anti-cancer therapy is one of the major challenges during treatment of multiple cancers. Gemcitabine is a standard first-line chemotherapeutic drug, yet autophagy is highly activated in the hypoxic microenvironment of solid tumors and enhances the survival of tumor cells against gemcitabine chemotherapy. Recently, we showed the add-on effect of autophagy inhibitor UAMC-2526 to prevent HT-29 colorectal tumor growth in CD1^-/-^ Foxn1nu mice treated with oxaliplatin. In this study, we aimed to investigate the potential beneficial effects of UAMC-2526 in a syngeneic Panc02 mouse model of pancreatic ductal adenocarcinoma (PDAC). Our data showed that UAMC-2526 combined with gemcitabine significantly reduced tumor growth as compared to the individual treatments. However, in contrast to *in vitro* experiments with Panc02 cells in culture, we were unable to detect autophagy inhibition by UAMC-2526 in Panc02 tumor tissue, neither *via* western blot analysis of autophagy markers LC3 and p62, nor by transmission electron microscopy. *In vitro* experiments revealed that UAMC-2526 enhances the potential of gemcitabine to inhibit Panc02 cell proliferation without obvious induction of cell death. Altogether, we conclude that although the combination treatment of UAMC-2526 with gemcitabine did not inhibit autophagy in the Panc02 mouse model, it has a beneficial effect on tumor growth inhibition.

## Introduction

Pancreatic ductal adenocarcinoma (PDAC) has become the second most common cause of cancer death in Western countries with an average survival rate below 6% (1, 2). The poor prognosis of this disease is due to the lack of early detection and resistance to current therapeutics (3, 4).

PDAC is characterized by a hypoxic microenvironment, as hypoxia inducible factor 1 (HIF-1) is highly expressed in 88% of pancreatic cancer tissue (5). Although gemcitabine has been approved by the FDA as first-line treatment of PDAC (6, 7), intrinsic and acquired resistance to gemcitabine is common. Several cell death pathways including macroautophagy (hereafter referred to as autophagy), necroptosis and apoptosis are involved in the pathogenesis of PDAC as well as in the responses and resistance mechanisms to gemcitabine-induced cytotoxicity (8, 9). Given that autophagy is induced by hypoxia, thereby negatively influencing the efficacy of chemotherapy, blocking this process may be a promising strategy in this highly mortal tumor type (9).

Autophagy is a highly conserved intracellular process by which double membranous vacuoles, known as autophagosomes, engulf cytoplasmic material including organelles and macromolecules for bulk degradation in lysosomes. This pathway plays a broad homeostatic role in intracellular housekeeping of the organism (10–12), and operates in cellular metabolism, cell death and survival (13, 14). Moreover, several studies have shown that autophagy is important for the prevention of a wide range of human pathological conditions such as cardiovascular disease, neurodegeneration, and numerous types of cancer (15–17).

The dynamic role of autophagy in cancer is dependent upon the stage of cancer progression. It can either repress the initial steps of carcinogenesis by suppressing chronic tissue damage, inflammation, and genome instability. On the other hand, autophagy is associated with cancer progression by sustaining energy production and by promoting cell survival in later stages (15, 18–20). Indeed, autophagy is generally recognized to function as a key survival mechanism that protects tumor cells exposed to cytotoxic stress. Several preclinical studies have shown very promising results by inhibiting autophagy as a novel target for cancer treatment (9, 21, 22).

Autophagy inhibition increases sensitivity to cytotoxic therapy and promotes cell death, both *in vitro* and *in vivo*. Currently available autophagy blockers such as chloroquine, 3-methyladenine (3-MA) and wortmannin are highly unspecific and lack potency so that severe toxicity is a common limitation to their use (23–27). Autophagy related 4B cysteine protease (ATG4B) converts pro-LC3 to LC3-I by cleaving its C-terminus after which LC3-I is conjugated to phosphatidylethanolamine by other ATGs, thereby forming LC3-II (28). Given the important role of this enzyme in autophagy and the encouraging results following ATG4B inhibition in several tumors, ATG4B is becoming increasingly attractive as a therapeutic target for cancer (24, 29–32).

We have previously shown that compound UAMC-2526, a low micromolar ATG4B inhibitor, inhibits tumor growth in a mouse HT-29 tumor xenograft model of human colorectal cancer by inhibiting ATG4B (33). In this model, UAMC-2526 significantly increased the therapeutic response to the cytostatic drug oxaliplatin. The latter is part of the clinical standard of care in advanced colorectal cancer. Moreover, UAMC-2526 did not cause any observable toxicity during the 28-day treatment regimen in this study. A safety pharmacology study for UAMC-2526 on 50 potential off-targets also did not reveal any issues that would preclude the further development of this compound as a drug candidate in oncology (34). Therefore, the aim of the present study was to further explore the beneficial effects of UAMC-2526 in the syngeneic Panc02 mouse model treated with gemcitabine.

## Material and Methods

### Cell Culture

Panc02 cells (murine ductal pancreatic adenocarcinoma) were grown in RPMI 1640 medium supplemented with 10% heat inactivated fetal bovine serum (FBS), 2 mM glutamine, 1% sodium pyruvate and 1% penicillin/streptomycin (Gibco, Life Technologies). Cells were incubated overnight at a density of 60,000 cells per well in 12-well plates at 37°C in 95% air/5% CO_2_ until confluency was reached. Hereafter, cells were treated with UAMC-2526 (10 µM) in the absence or presence of gemcitabine (10 µM) for 32 hours. For measuring the autophagy flux 160 nM of bafilomycin A_1_ (Santa Cruz Biotechnology, sc-2021550) was added for 2 hours.

### *In Vitro* Proliferation and Cell Death Assay

Panc02 cells were incubated overnight at a density of 60,000 cells per well in 12-well plates after which UAMC-2526 was added at different concentrations in the presence or absence of 10 µM gemcitabine. Cell proliferation was measured using neutral red solution as previously described (35). Briefly, cells were incubated for 2 hours with 0.1% neutral red solution at 37°C in 95% air/5% CO_2_. Subsequently, cells were washed with PBS and neutral red was extracted by addition of 0.05 M NaH_2_PO_4_ in 50% ethanol. After 3 minutes, optical density was read at 540 nm using a microtiter plate reader.

To detect cell death, 1 µg/ml propidium iodide (PI, Molecular Probes) and 10 µg/ml Hoechst (Life Technologies) was added, followed by visualization of PI/Hoechst-labeled cells using a Celena S digital Imaging System (Logos Biosystems).

### *In Vivo* Panc02 Tumor Model

4×10^6^ viable Panc02 cells (suspended in 100μl PBS) were injected into the right hind leg of female C57/BL6 mice (n= 40, 6-7 weeks old; Charles River Laboratories). All animals received a numerical code throughout the whole experiment and were kept under environmentally controlled conditions (12 hours light/dark cycle, 20–24°C and 40–70% relative humidity) with food and water ad libitum. When tumors reached an approximate volume of 150 mm^3^, mice were randomly divided into 4 groups (10 mice per group) for treatment with clinical-grade gemcitabine (Selleck Chemicals), UAMC-2526, gemcitabine combined with UAMC-2526, or vehicle (DMSO/propylene glycol/ethanol 50:40:10). Gemcitabine (60 mg.kg^-1^) was administered i.p. 3x/week for 28 days. Unidose solutions of compound UAMC-2526 were prepared in vehicle according to the individual mouse weight and loaded into snap-fit-sealed polypropylene microvials. Solutions were prepared the day before treatment and transferred to osmotic minipumps (model 1002, Alzet) for 2-week applications achieving an *in vivo* pumping rate of 3 mg.kg^-1^.day^-1^. The control and gemcitabine group received vehicle administered *via* a 100 µl osmotic minipump system. The osmotic minipumps were replaced after 2 weeks to obtain a total treatment period of 28 days. Mice not treated with gemcitabine received an i.p. injection with saline at the same time.

Body weight was measured three times a week and tumor growth was monitored three times a week with digital caliper measurements starting upon detection of palpable tumors. Tumor volume was calculated using the modified ellipsoid formula [i.e., ½ (length×width^2^)]. Relative tumor volume (RTV) was calculated as V_x_/V_1_, with V_x_, volume at time point t, and V_1_, the volume at baseline. The percentage of tumor growth inhibition (TGI) was calculated as 1- (RTVx/RTVc)x100 with RTVx being the relative tumor volume from the treated group and RTVc the relative tumor volume from the control group. The tumor doubling time was calculated as V= V_0_2^t/d^. Tumor samples were isolated on day 28 or when tumors reached a volume of 1500 mm^3^. The study was conducted in accordance with the Declaration of Helsinki and was approved by the ethics committee of the University of Antwerp (file number 2017-68).

### PET/CT Imaging

^18^F-FDG was produced in an automated module of the Department of Radiopharmacy at Antwerp University Hospital using a FASTlab FDG Cassette (GE Healthcare). Small animal PET imaging was performed using two Siemens Inveon PET-CT scanners (Siemens Preclinical Solution). The acquired PET data were reconstructed using 4 iterations with 16 subsets of the 2D ordered subset expectation maximization (OSEM 2D) algorithm following Fourier rebinning (FORE). Normalization, dead time, random CT-based attenuation and single-scatter stimulation (SSS) scatter corrections were applied. CT voltage and amperage were set to 80 keV and 500 μA, respectively. Animals received two PET scans: one baseline (before start of the treatment) and one follow-up PET scan, 6-7 days after start of the treatment. Mice first received an IV bolus injection of ^18^F-FDG (37 MBq) in the left lateral tail vein, immediately followed by a double measurement (Easy-Touch, Lifescan, France) of pre-scan whole blood glucose levels, from a drop of blood collected from the contralateral tail vein. The average value was considered to correct for blood glucose levels. Twenty-five minutes after the ^18^F-FDG injection (i.e. uptake period), mice were anesthetized with isoflurane (5% for induction and 2% for maintenance) and five minutes after the start of the anesthesia mice were prepared and positioned onto the PET scanner, resulting in a total uptake period of 30 min. Sequentially to the PET scan, a 7 min CT scan was performed for attenuation as well as scatter correction purposes and for anatomical localization of the tumors. To minimize variability, all mice were handled under the same anesthetic agent, incubation time, uptake period and body temperature. Mice were not fasted as fasting can severely induce autophagy. Several quantification methods were applied to quantify ^18^F-FDG uptake in the tumors. Regions of interest (ROIs) were drawn on the CT images manually by qualitative assessment covering the whole tumor slice-by-slice. Tumor volume and tracer uptake were generated by summation of voxels within the tomographic planes using PMOD Software 6.3 (PMOD technologies) and expressed by mean and max standardized uptake values (SUVmean or SUVmax). The standardized uptake value (SUV) was thereby defined as (CT×W)/Dinj, where CT is radioactivity in the tissue (kBq/cc), W is weight of the animal (g) and Dinj is injected dose (kBq). SUVmean represents the mean concentration of the tracer and was calculated with and without correction for the glucose (Glc) level: (CT×W×Glc)/Dinj. SUV max represents the concentration in the 5 hottest pixels. In addition, total lesion glycolysis (TLG) was calculated by multiplying the SUVmean and the tumor metabolic volume (TMV). A threshold method was also applied to quantify tracer tumor uptake on the PET image, whereby an elliptic volume-of-interest that enclosed the entire tumor was positioned manually and was centered on the tumor area that showed maximal radiotracer uptake. Then 3-dimensional isocontours at 60% of the maximum pixel value within this volume-of-interest were drawn automatically.

### Immunohistochemistry

Tissue samples were fixed in 4% neutral buffered formalin (Sigma-Aldrich) for 24 hours prior to paraffin embedding. To assess tumor cell proliferation, tumor sections (5 µm thick) were incubated with primary antibodies against MCM2 (ab108935). Besides immunohistochemistry, tumor sections were also stained with hematoxylin-eosin and acellular areas histologically analyzed to quantify the percentage of tumor necrosis. Apoptosis was determined *via* TUNEL assay using the ApopTag Plus Peroxidase *In situ* Apoptosis kit (Merck, S7101). All images were acquired with an Olympus U-TUIX-2 microscope and analyzed with ImageJ 1.42 software (National Institutes of Health). Analyses were carried out without prior knowledge of the treatments.

### Transmission Electron Microscopy

Tumor samples were prepared for TEM analysis as previously described (36). A FEI Tecnai microscope was used to examine ultrathin sections at 80-120 kV. Autophagic vacuoles were quantified on 40 different images taken at random per sample.

### Western Blotting

Tumor samples were mixed in RIPA lysis buffer (Sigma-Aldrich, R0278) containing protease and phosphatase inhibitors (Roche Diagnostics, 046931590001; 049606845001) using a Percellys 24 tissue homogenizer (Bertin Instrument). After centrifugation, the protein concentration of the supernatant of each sample was determined using the bicinchoninic acid method (BCA, ThermoFischer). Laemmli buffer (Bio-Rad, 1610737) containing 5% β-mercaptoethanol (Sigma-Aldrich) was added and samples were heat-denatured for 5 minutes in boiling water. Cell culture samples were directly lysed in Laemmli sample buffer (Bio-Rad, 1610737) containing 5% β-mercaptoethanol (Sigma-Aldrich) and heat-denatured for 5 min in boiling water. Next, equal amounts of protein were separated on pre-cast Novex Bolt 12% Bis-Tris gels (Invitrogen, NW00125BOX) and blotted onto Immobilion-FL polyvinylidene fluoride membranes. After incubation in Odyssey^®^ Blocking Buffer (Li-Cor Biosciences, LI 927-50000), membranes were probed overnight at 4°C with primary antibodies diluted in Odyssey Blocking Buffer, followed by a one hour incubation with IRDye-labeled secondary antibodies at room temperature. Antibody detection was achieved using an Odyssey SA infrared imaging system (LI-COR Biosciences). The intensity of the protein bands was quantified using Emperia software. Primary antibodies included anti-p62/SQSTM1 (Abcam, ab56416), anti-LC3 (Nanotools, 0231-100/LC3-5F10) and anti-ACTB (Abcam, ab115777). IRDye-labeled secondary antibodies (goat anti-mouse [IgG926-68070] and goat anti-rabbit [IgG926-32211]) were purchased from LI-COR Biosciences.

### Statistical Analyses

Results are expressed as mean ± SEM. All statistical analyses were performed using SPSS software (version 27, SPSS Inc). Statistical tests are specified in the figure legends. Differences were considered significant at p ≤ 0.05.

## Results

### UAMC-2526 Inhibits Proliferation of Panc02 Pancreatic Carcinoma Cells Without Inducing Cell Death

**Figure 1:**
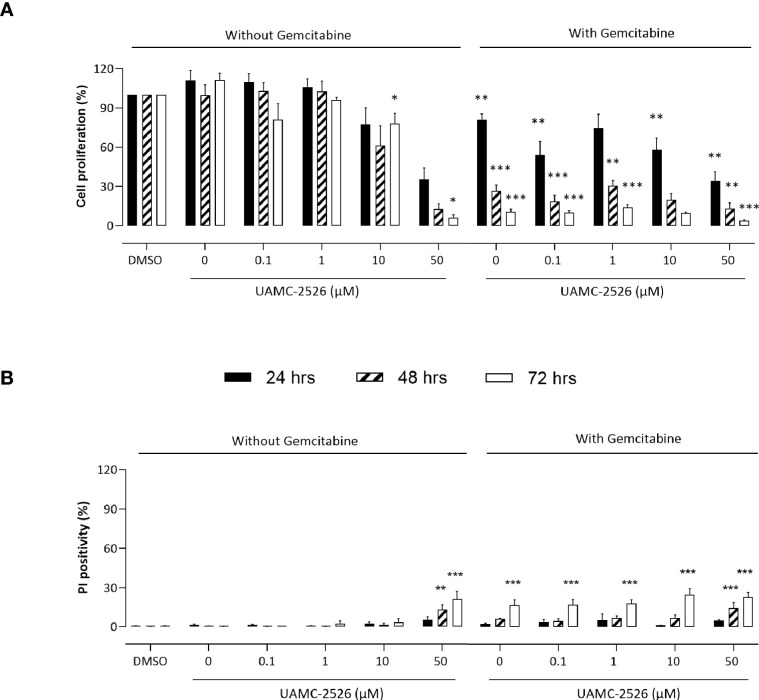
UAMC-2526 inhibits Panc02 cell proliferation. **(A)** Panc02 cells were treated *in vitro* with different concentrations of UAMC-2526 in the presence or absence of gemcitabine (10 µM). Cell proliferation was measured at different time points with neutral red. Data were analyzed using one sample t-test. Six independent experiments were performed. After normalization, the resulting fold change was log transformed (log2-base) to obtain an approximately normal distribution. *p ≤ 0.05, **p ≤ 0.01, ***p ≤ 0.001 *versus* DMSO group. **(B)** Panc02 cells were treated *in vitro* with different concentrations of UAMC-2526 in the presence or absence of gemcitabine (10 µM). Cell death was measured at different time points by propidium iodide (PI) labeling. Five independent experiments were performed. Two-way ANOVA followed by Dunnett’s *post hoc* test. **p ≤ 0.01, ***p ≤ 0.001 *versus* DMSO group.

To investigate the potential beneficial effects of UAMC-2526, Panc02 cells were treated *in vitro* with UAMC-2626 in the presence or absence of gemcitabine. Cell proliferation was inhibited by UAMC-2526 in a concentration and time dependent way (**Figure 1A**). Combined treatment of Panc02 cells with UAMC-2526 and gemcitabine almost completely prevented cell proliferation. To determine whether the inhibition of cell proliferation was related to cell death induction, propidium iodide (PI) labeling experiments were performed (**Figure 1B**). UAMC-2526 did not induce cell death in a concentration range between 0.1-10 µM. However, administration of UAMC-2526 at a higher concentration (50 µM) combined with longer incubation times (48 and 72 hours) showed a significant increase in PI positive cells, even though the number of PI positive cells remained limited (<25%). Gemcitabine (10 µM) initiated cell death after 72 hours of treatment. Combined treatment of Panc02 cells with UAMC-2526 and gemcitabine showed an additive, but no synergistic effect on cell death induction.

### UAMC-2526 Inhibits LC3 Processing in Panc02 Cells

**Figure 2:**
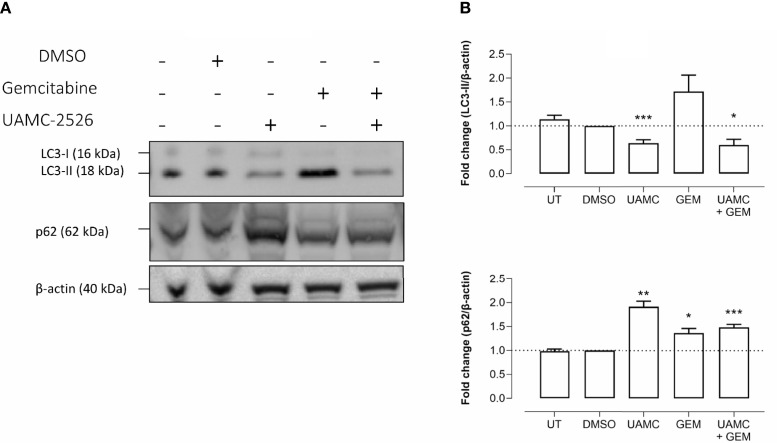
UAMC-2526 inhibits LC3 processing in Panc02 cells. **(A)** Panc02 cells were treated *in vitro* with UAMC-2526 (UAMC, 10 µM) in the presence or absence of gemcitabine (GEM, 10 µM) for 32 hours. Western blots were performed for LC3-II and p62. β-actin served as a loading control. One representative blot (out of five independent experiments) is shown. **(B)** Quantification of LC3-II and p62 relative to β-actin. Data were analyzed using one sample t-test. After normalization, the resulting fold change was log transformed (log2-base) to obtain an approximately normal distribution,*p ≤ 0.05, **p ≤ 0.01***p ≤ 0. 001, *versus* DMSO group. UT, untreated.

**Figure 3:**
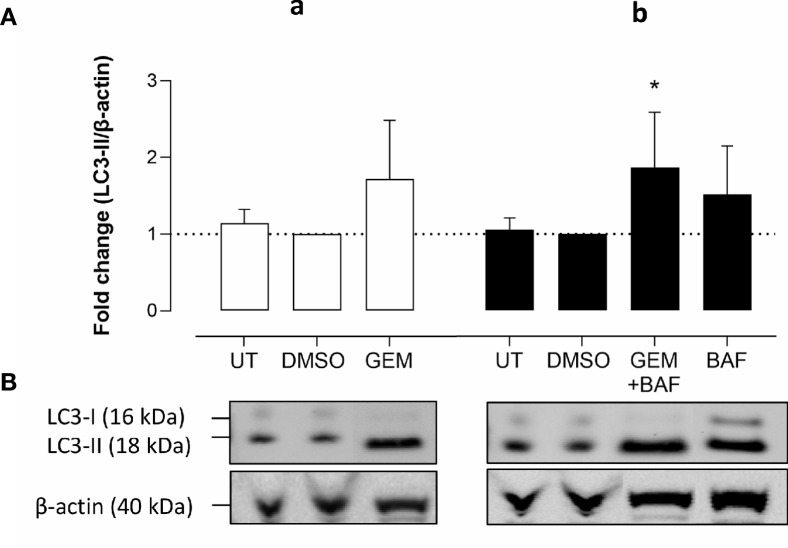
Gemcitabine does not induce autophagy flux. **(A-a)** Panc02 cells were treated *in vitro* with gemcitabine (GEM, 10 µM) for 32 hours. **(A-b)** For measuring autophagy flux in gemcitabine condition, bafilomycin A_1_ (BAF, 160 nM) was added in the last two hours. Western blots were performed for LC3-II. β-actin served as a loading control. LC3-II was quantified relative to β-actin. Data were analyzed using one sample t-test. After normalization, the resulting fold change was log transformed (log2-base) to obtain an approximately normal distribution,*p ≤ 0.05 *versus* DMSO group. UT, untreated. **(B)** One representative blot (out of five independent experiments) is shown.

Western blots were performed on cell lysates of Panc02 cells to analyze the expression of autophagy marker LC3-II. Administration of UAMC-2526 decreased basal autophagy in Panc02 cells as shown by a decreased conversion of the soluble isoform LC3-I into the autophagosome-associated isoform LC3-II. Gemcitabine treatment initiated autophagosome formation, as demonstrated by elevated levels of LC3-II, but this effect was significantly blunted in the presence of UAMC-2526 (**Figure 2**). Moreover, both UAMC-2526 and gemcitabine stimulated p62 accumulation (**Figure 2**). Inhibition of autophagic flux by adding bafilomycin A1 blocked LC3-II degradation and allowed the accumulation of LC3-II in untreated cells. However, bafilomycin A1 did not further stimulate LC3-II levels in gemcitabine-treated cells (**Figure 3**).

### UAMC-2526 Potentiates Chemotherapy Efficiency

**Figure 4:**
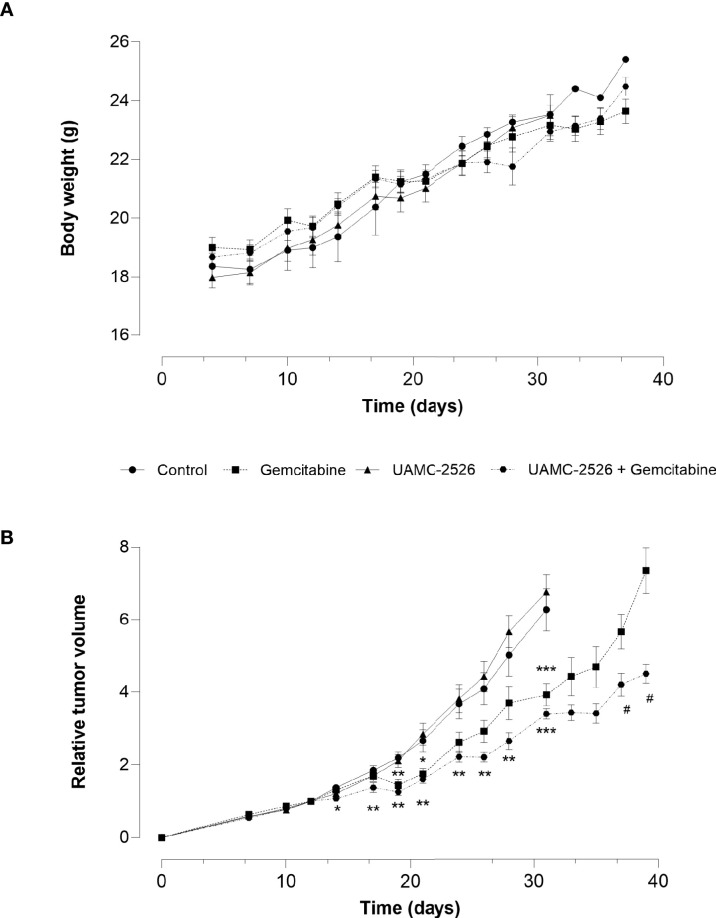
Effect of UAMC-2526 on Panc02 tumor growth in C57/BL6 mice. Animals were injected with 4×10^6^ Panc02 cells. When tumors reached an approximate volume of 150 mm^3^, mice were treated with vehicle (control), UAMC-2526, gemcitabine or a combination of UAMC-2526 and gemcitabine. Number of animals in each group was between 8-10. **(A)** Treatments had no effect on body weight. **(B)** Effects of treatment on relative tumor volumes (RTV). RTV values from D0 to D31 were examined with one-way ANOVA. Significant values of *post hoc* comparison were adjusted using Dunnett’s correction. *p ≤ 0.05, **p ≤ 0.01, ***p ≤ 0.001 *versus* control group. Because tumor development in the control group and UAMC-2526 treated group was too extensive, these animals were sacrificed at D31. RTV values of the remaining groups were examined using Welch’s t-test. ^#^p ≤ 0.05 *versus* monotreatment with gemcitabine.

The effect of UAMC-2526 on tumor growth was further explored *in vivo* using C57/BL6 mice inoculated with Panc02 cells. When tumors reached an approximate volume of 150 mm^3^ (D0), mice were randomly divided into four treatment groups: vehicle, gemcitabine, UAMC-2526 and UAMC-2526 combined with gemcitabine. Body weight did not differ during the treatment between groups (**Figure 4A**), albeit relative tumor volume (RTV) rapidly increased in the vehicle and UAMC-2526 treated groups from D14 (**Figure 4B**). Compared to vehicle treated controls, mice treated with gemcitabine showed a reduced RTV from D19. Combined administration of gemcitabine and UAMC-2526 further reduced RTV compared to vehicle and monotreatment with gemcitabine from D14.

**Figure 5:**
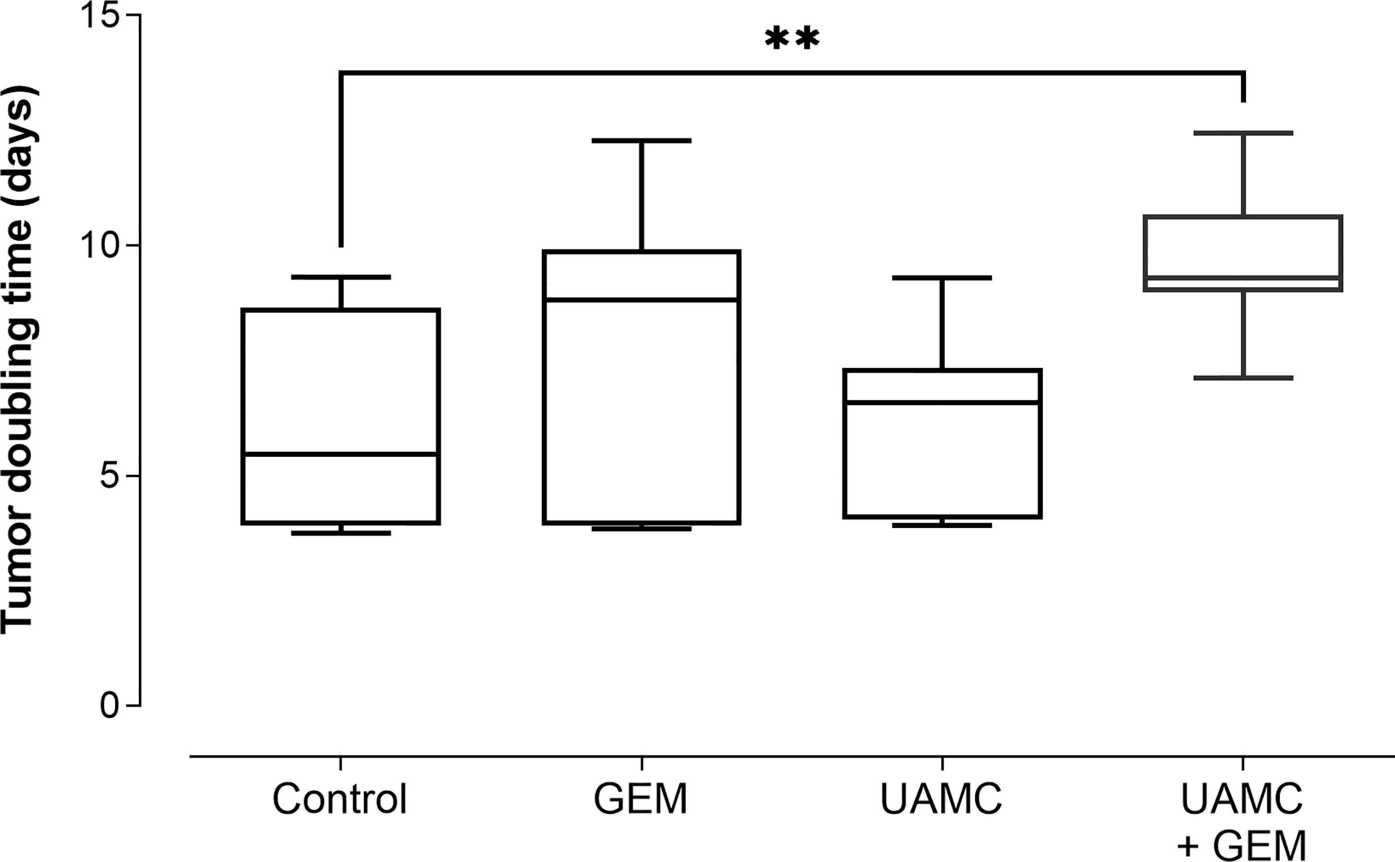
Tumor doubling time. C57/BL6 mice with Panc02 tumors were treated with vehicle (control), UAMC-2526 (UAMC), gemcitabine (GEM) or a combination of UAMC-2526 and gemcitabine. After treatment, tumor doubling time was calculated. Number of animals in each group was between 8-10. Data were analyzed with one-way ANOVA. Significant values of *post hoc* comparison were adjusted using Dunnett’s correction. **p ≤ 0.01 *versus* control group.

Next to RTV, the percentage tumor growth inhibition (%TGI) was calculated (**Table 1**). Administration of gemcitabine led to an average %TGI of 27.4 ± 4.0 between D24-D31, while combined treatment with UAMC-2526 resulted in a significantly higher average %TGI of 44.5 ± 1.9 (***p ≤ 0.001, Dunnett’s T3 Multiple Comparison Test, n = 39-40). In addition, tumor doubling time increased significantly after combined treatment with gemcitabine and UAMC-2526 (**Figure 5**).

**Table 1:** Percentage tumor growth inhibition (%TGI) of Panc02 tumors after 24-31 days (D24-D31) of treatment.

Treatment	%TGI D24	%TGI D26	%TGI D28	%TGI D31
Gemcitabine	28.7 ± 7.7**	28.5 ± 7.4**	26.2 ± 7.4*	37.3 ± 4.9***
UAMC-2526	-3.9 ± 10.5	-8.2 ± 10.1	-12.8 ± 8.7	-7.7 ± 7.7
UAMC-2526 Gemcitabine	39.4 ± 4.2***	45.8 ± 3.4***	47.1 ± 4.6***	45.7 ± 2.3***

Data were analyzed with one-sample t-test. *p ≤ 0.05, **p ≤ 0.01, ***p ≤ 0.001 versus 0 (n=4-10).

### 18F-FDG PET Does Not Demonstrate Differences in Metabolic Activity in Treated Panc02 Tumors

**Figure 6:**
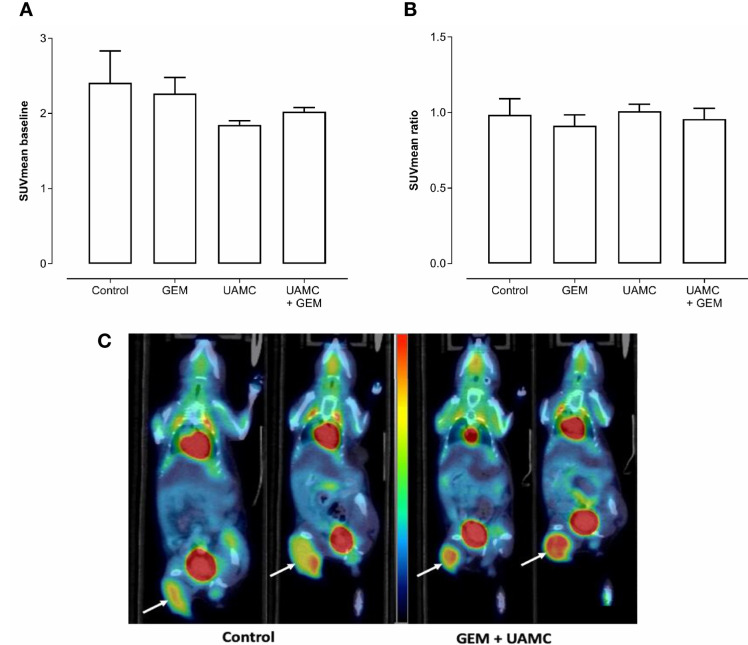
PET/CT imaging of Panc02 tumors. C57/BL6 mice bearing Panc02 tumors were treated with vehicle (control), UAMC-2526 (UAMC), gemcitabine (GEM) or a combination of UAMC-2526 and gemcitabine. **(A)** Mice underwent a ^18^F-FDG PET scan before the start of the treatment (baseline) and SUVmean was calculated. Number of animals in each group was between 4-7. Data were analyzed with factorial analysis of variance (ANOVA). No significance was found with factors GEM (p = 0.945) and UAMC (p = 0.108), interaction GEM by UAMC (p = 0.503). **(B)** Mice underwent a ^18^F-FDG PET scan after 6-7 days of treatment. Thereafter, the SUVmean ratio *versus* baseline was calculated for the different treatment groups. Number of animals in each group was between 4-7. Data were analyzed with ANOVA. No significance was found with factors GEM (p = 0.445) and UAMC (p = 0.665), interaction GEM by UAMC (p = 0.899). **(C)** Representative ^18^F-FDG PET/CT images at baseline and after 6-7 days of treatment. White arrows indicate the tumor. One mouse of the control group scanned at baseline (left) and after 6-7 days of treatment (right). One mouse of the combination group receiving gemcitabine and UAMC compound, also scanned at baseline (left) and after 6-7 days of treatment (right). All images were scaled to the same color scale (SUV scale from 0 – 4).

According to PET/CT imaging, all treatment groups showed a clear ^18^F-FDG uptake with no significant differences between groups at baseline (i.e. before treatment; **Figure 6A**). After 6-7 days of treatment, no significant differences in ^18^F-FDG uptake between treatment groups could be observed (irrespective of the PET quantification method, **Figure 6B**). None of the quantification methods showed significant results. Also a correction for blood glucose levels did not change significances. Representative PET/CT images are shown in **Figure 6C**.

### UAMC-2526 Has No Impact on Autophagy in Panc02 Tumors

**Figure 7:**
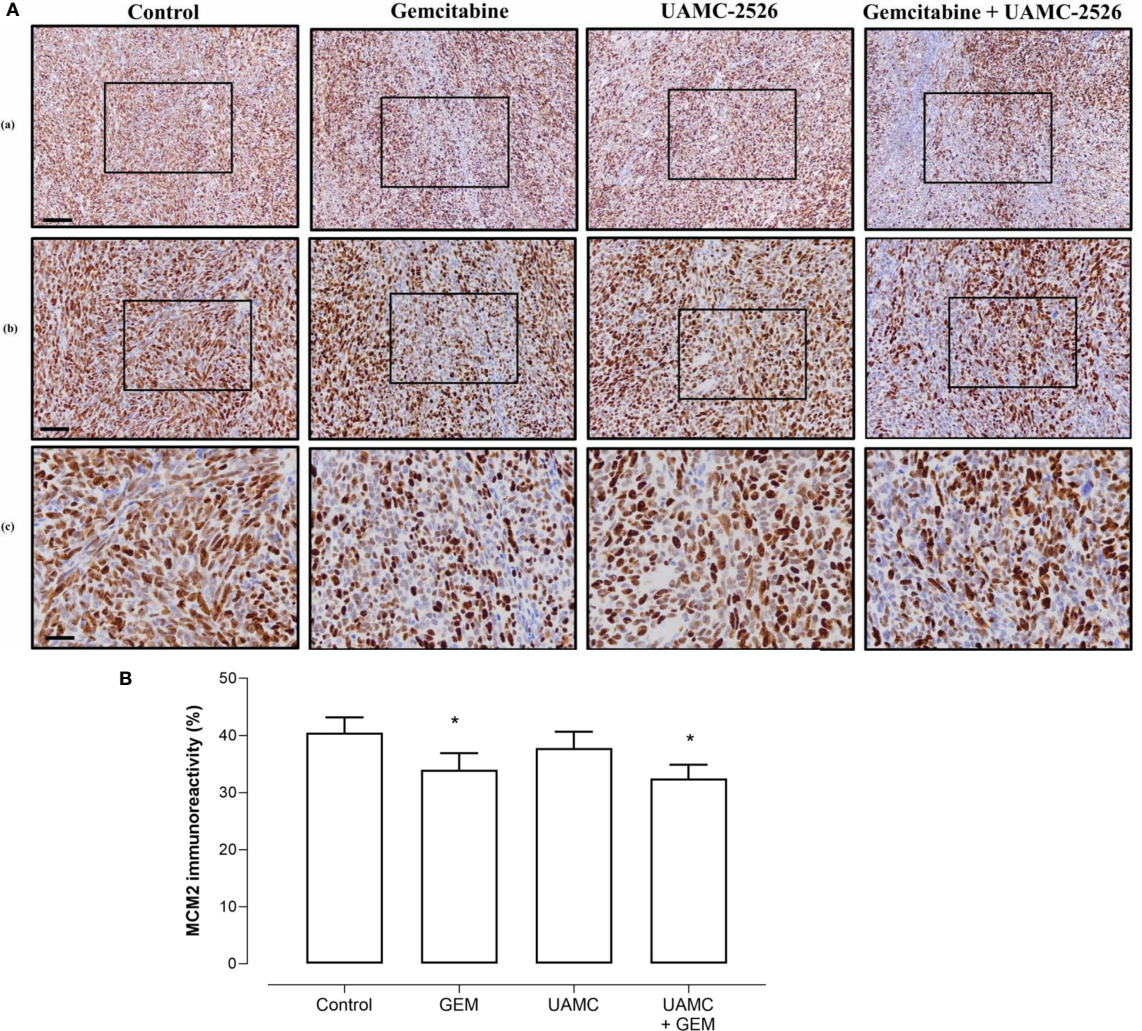
Immunohistochemical analysis of cell proliferation marker MCM2 in Panc02 tumors. **(A)** C57/BL6 mice with Panc02 tumors were treated with vehicle (control), UAMC-2526 (UAMC), gemcitabine (GEM) or a combination of UAMC-2526 and gemcitabine. After treatment, tissue sections of Panc02 tumors were immunostained with MCM2 antibodies. Scale bar = 100 µm (a), 50 µm (b), 20 µm (c). **(B)** Quantification of MCM2 immunoreactivity. Number of animals in each group was between 8-10. Data were analyzed with factorial analysis of variance (ANOVA), *p ≤ 0.05 with factors GEM (p=0.041) and UAMC (p=0.452), interaction GEM by UAMC (p=0.840).

**Figure 8:**
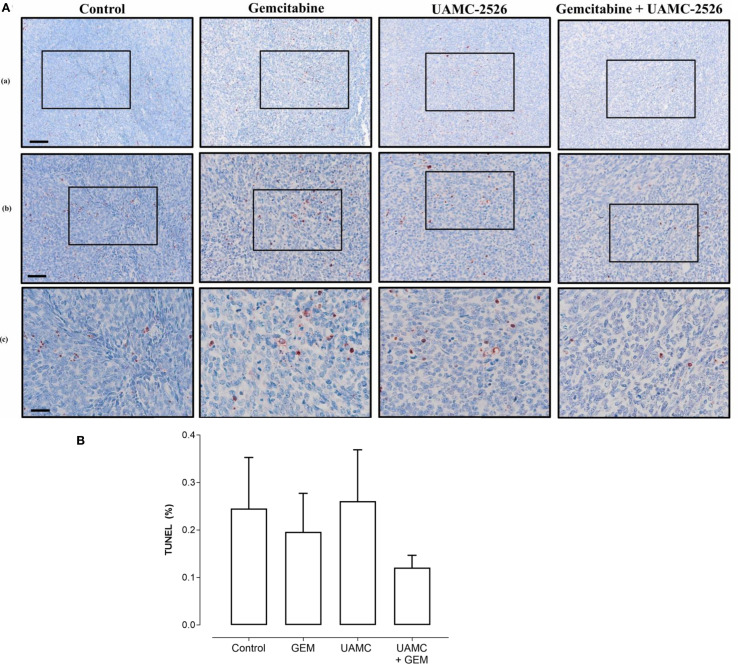
Histological analysis of apoptosis in Panc02 tumors. **(A)** C57/BL6 mice with Panc02 tumors were treated with vehicle (control), UAMC-2526 (UAMC), gemcitabine (GEM) or a combi-nation of UAMC-2526 and gemcitabine. After treatment, apoptotic cells were detected in tis-sue sections of Panc02 tumors via TUNEL assay. Scale bar = 100 µm (a), 50 µm (b), 20 µm (c). **(B)** Quantification of TUNEL. Number of animals in each group was between 8-10. Data were analyzed with factorial analysis of variance (ANOVA), no significance was found with factors GEM (p=0.321) and UAMC (p=0.750), interaction GEM by UAMC (p=0.631). Scale bar = 100 µm (a), 50 µm (b), 20 µm (c).

**Figure 9:**
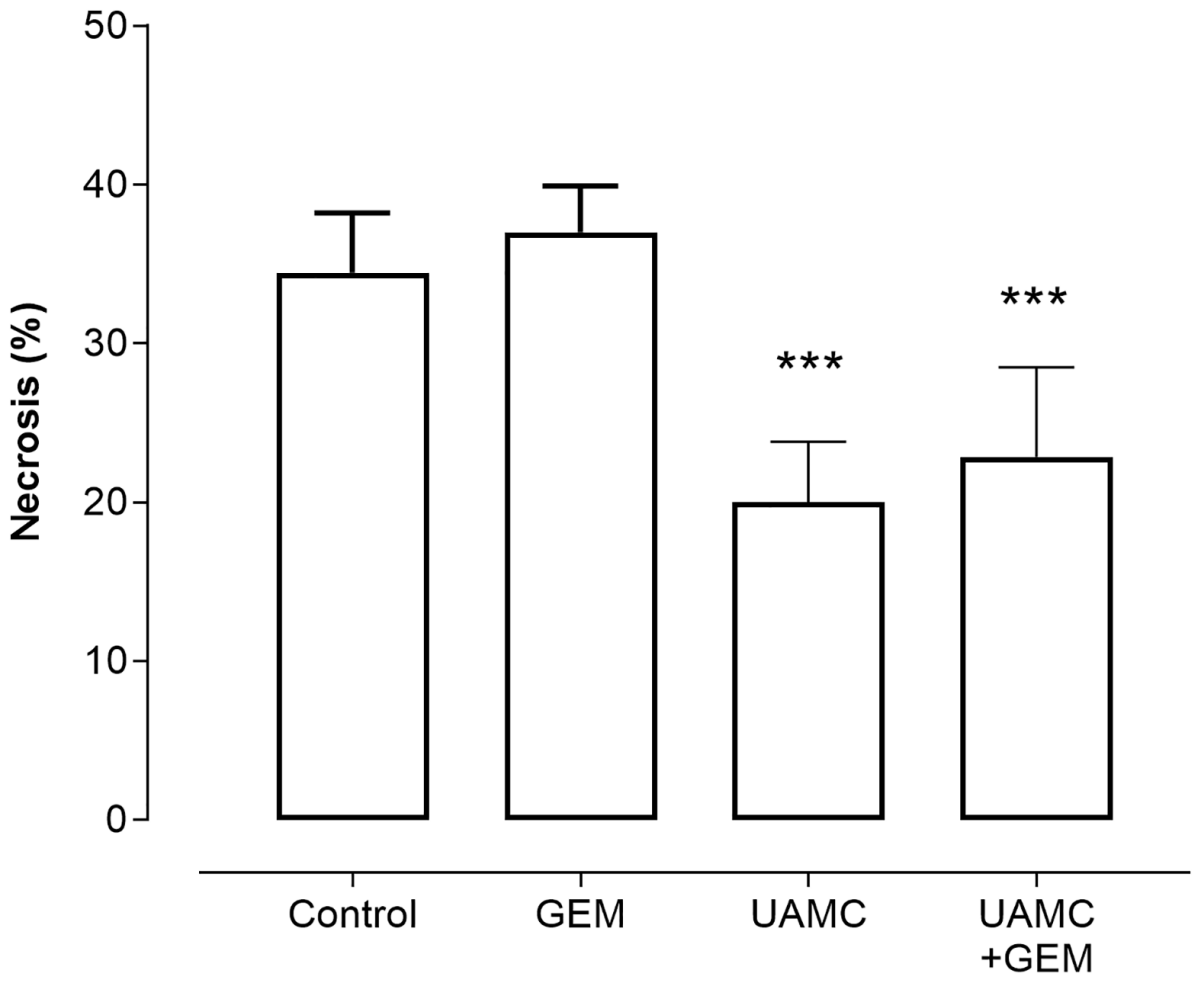
Histological analysis of necrosis in Panc02 tumors. C57/BL6 mice with Panc02 tumors were treated with vehicle (control), UAMC-2526 (UAMC), gemcitabine (GEM) or a combination of UAMC-2526 and gemcitabine. After treatment samples were immunostained with hematoxylin-eosin. Number of animals in each group was between 8-10. Data were analyzed with factorial analysis of variance (ANOVA), ***p ≤ 0.001 with factors UAMC (p=0.001), GEM (p=0.499) and interaction UAMC by GEM (p=0.970).

**Figure 10:**
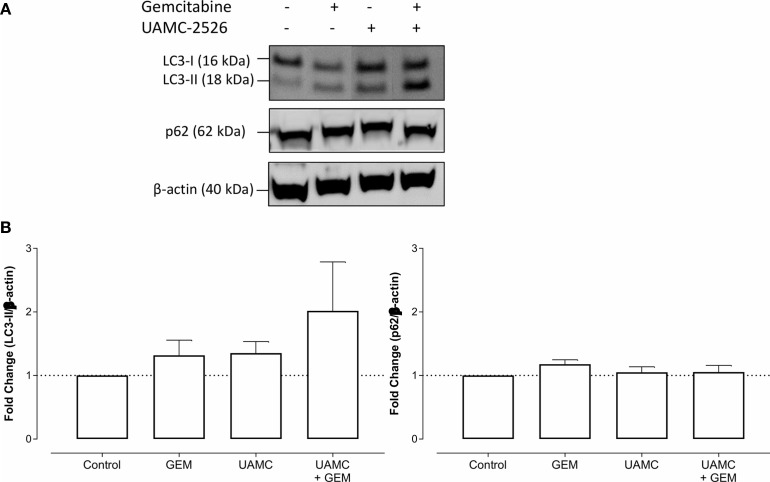
UAMC-2526 has no impact on autophagy in Panc02 tumors. **(A)** C57/BL6 mice with Panc02 tumors were treated with vehicle (control), UAMC-2526 (UAMC), gemcitabine (GEM) or a combination of UAMC-2526 and gemcitabine. After treatment, western blots were performed on Panc02 tumor lysates to detect LC3-II and p62. β-actin served as a loading control. Representative images are shown. **(B)** Quantification of LC3-II and p62 relative to β-actin. Data were analyzed using one-sample t-test. After normalization, the resulting fold change was log transformed (log2-base) to obtain an approximately normal distribution. Number of animals in each group was between 8-10. No significance was demonstrated.

**Figure 11:**
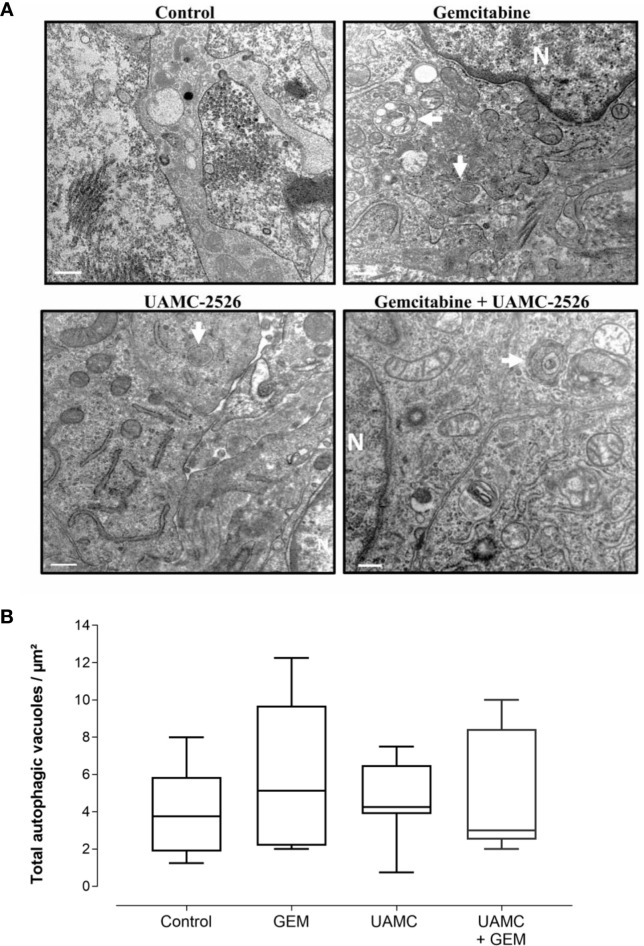
Detection of autophagy in Panc02 tumors via transmission electron microscopy. **(A)** C57/BL6 mice with Panc02 tumors were treated with vehicle (control), UAMC-2526 (UAMC), gemcitabine (GEM) or a combination of UAMC-2526 and gemcitabine. After treatment, ultrathin sections of Panc02 tumors were analyzed for the presence of autophagic vacuoles (white arrows) using transmission electron microscopy. Representative images are shown, Scale bar = 500 nm. N = nucleus. **(B)** Quantification of autophagic vacuoles. Number of animals in each group was between 8-10. One-way ANOVA test showed no significance.

Microscopic analysis of gemcitabine-treated tumor samples revealed a modest, albeit significant reduction in immunoreactivity for cell proliferation marker MCM2 as compared to vehicle-treated controls (**Figure 7**). Also tumors of mice treated with UAMC-2526 and gemcitabine showed a significant decrease in immunoreactivity for MCM2 as compared to the control group. However, there was no significant difference in MCM2 immunostaining in tumors of UAMC-2526 treated mice. The frequency of tumor apoptosis between treated animals and controls did not change (**Figure 8**), though there was a significant reduction of necrosis in tumors of mice treated with UAMC-2526 (**Figure 9**). Western blots were performed on lysates of tumor samples to analyze the autophagosomal marker protein LC3-II and selective autophagy receptor p62 (**Figure 10**). Neither LC3-II nor p62 levels were significantly different between groups. Similarly, electron microscopy did not show any significant differences in the formation of autophagic vacuoles in tumor tissues of the combination treatment group as compared to the controls (**Figure 11**).

## Discussion

Current therapies for PDAC have limited success due to therapeutic resistance (37). The microenvironment of cancers plays a major role in the resistance mechanism by influencing different important cellular processes such as proliferation, invasion of tumor cells, chemosensitivity, angiogenesis and tumorigenesis. PDAC is characterized by a hypoxic microenvironment as evidenced by high HIF-1 expression rates in the pancreatic cancer tissue (38). Interestingly, several studies have demonstrated that PDAC cells require autophagy to proliferate and to survive in this hypoxic condition. Increased levels of autophagy activity in these cells correlates with poor patient outcome (39, 40). Inhibition of autophagy, on the other hand, has been shown to restore sensitivity to cytotoxic therapy and to inhibit tumor growth (9, 41). We have previously shown that compound UAMC-2526 inhibits tumor growth in a mouse HT-29 tumor xenograft model of human colorectal cancer by inhibiting cysteine protease ATG4B (33). Several studies have demonstrated that ATG4B plays an important role in various cancers (30–33). Increased expression levels of ATG4B are associated with both tumor progression and therapy resistance. Accordingly, ATG4B is a potential anti-cancer target and a valid alternative to anti-lysosomal therapy due to its role in LC3 processing and autophagosome formation (28).

There is strong evidence indicating that pancreatic cancer cells are vulnerable to inhibition of ATG4B (42). In the present study, we aimed to investigate the potential beneficial effects of UAMC-2526 in a Panc02 mouse model of PDAC treated with gemcitabine. For this purpose, we first examined the UAMC-2526 autophagy inhibitory effect on Panc02 cells *in vitro*. UAMC-2526 significantly decreased both basal autophagy of Panc02 cells and elevated levels of LC3-II induced by gemcitabine. Western blots also demonstrated a significant increase of p62 expression after treatment with UAMC-2526 and/or gemcitabine. Surprisingly, inhibition of autophagic flux by bafilomycin A1 did not further stimulate the elevated LC3-II levels in gemcitabine-treated cells. This finding suggests that gemcitabine stimulates autophagosome formation in Panc02 cells, but not autophagic flux. This may also explain the elevated p62 levels after gemcitabine treatment.

The next step was to assess these effects *in vivo*, using a mouse model bearing subcutaneous Panc02 tumors. Mice received either vehicle, UAMC-2526, the standard chemotherapy drug gemcitabine or a combination of UAMC-2526 and gemcitabine. To assess an early treatment response, we investigated whether PET imaging with the standard clinical radiotracer fluorodeoxyglucose (^18^F-FDG) could predict differences in tumor growth inhibition (TGI) after 1 week of treatment. ^18^F-FDG accumulates in tumor cells due to an increased reliance on glycolysis for energy production and is closely related to their proliferation rate (43, 44). However, no differences in ^18^F-FDG uptake could be observed in our model, despite a significant reduction of the tumor volumes after treatment with UAMC-2526 and gemcitabine at later time points. A possible explanation could be the timing of PET scanning, which could be either too late or too soon, or the nonspecific uptake of ^18^F-FDG.

The average TGI after 24-31 days of combined treatment with UAMC-2526 and gemcitabine was 44.5%, which is significantly higher as compared to the average TGI (27.4%) evoked by gemcitabine. Moreover, the tumor doubling time, which is the amount of time that a tumor takes to double in size, was significantly increased if treated with both UAMC-2526 and gemcitabine. Tumor growth was not inhibited after individual treatment with UAMC-2526. It should be noted, however, that although tumor tissues of mice treated with the combination UAMC-2526/gemcitabine showed a significant decrease of tumor growth as compared to the individual treatments (or the vehicle-treated group), we could not correlate the decreased tumor growth with autophagy inhibition as typical features of autophagy inhibition such as a decrease in LC3-II, accumulation of the selective autophagy receptor p62 or an impaired formation of autophagic vesicles could not be demonstrated.

Several issues should be addressed to explain the discrepancy between the *in vitro* and *in vivo* findings of the present study in terms of autophagy inhibition. Possibly, the dosage used was not high enough to inhibit the autophagy pathway in the Panc02 tumors. Because the plasma stability of UAMC-2526 is limited (33), we were forced to administer the compound *via* a subcutaneously implanted osmotic minipump to guarantee a continuous supply of fresh compound. However, also the maximal solubility of the compound within the osmotic minipumps is limited so that a dosage not higher than 3 mg.kg^-1^.day^-1^ could be applied. Furthermore, it should be noted that some autophagy modulating drugs may play multiple roles in the autophagy process and could react differently depending on the experimental conditions. For example, FMK-9a is the most potent ATG4B inhibitor reported thus far, but despite its strong potential to inhibit ATG4B activity, FMK-9a is able to induce autophagy in HeLa and MEF cells (45). Furthermore, intratumor heterogeneity, which refers to distinct tumor cell populations within the same tumor specimen, is associated with an incomplete response to therapy (46–48). All these arguments indicate that modulation of autophagy is cell type- and context-dependent. Prior to autophagy modulation it is highly important to consider the type and tumor staging of cancers as well as the involvement of autophagy in the particular cancer type.

If autophagy inhibition did not occur, what could be the mechanism behind the observed tumor growth inhibition? Given that ATG4B inhibitors with diverse structures are able to inhibit tumor growth (33, 49–51), it cannot be excluded that ATG4B inhibiting compounds may also affect other targets than ATG4B. Indeed, some studies indicate that autophagy inhibitors enhance the inhibition of cell proliferation (52, 53). *In vitro* data from the present study confirmed that Panc02 proliferation is significantly reduced by both UAMC-2526 and gemcitabine. Inhibition of Panc02 cell proliferation was most pronounced after combined treatment with UAMC-2526 and gemcitabine, indicating the add-on effect of UAMC-2526. In addition, the reduced proliferation was strongly time dependent. However, in contrast to gemcitabine, UAMC-2526 did not reduce the immunoreactivity for cell proliferation marker MCM2 in tumor samples. Two aspects should be considered that may explain these findings. Firstly, our *in vitro* experiments showed that gemcitabine is a more potent inhibitor of Panc02 cell proliferation as compared to UAMC-2526. Secondly, given that MCM2 is highly expressed in several types of cancers (including PDAC), and that MCM2 has a much longer half-life as compared to other proliferation markers (e.g. Ki-67), it might take more time before the inhibitory effects of UAMC-2526 on MCM2 expression are visible. It should be noted that, besides inhibition of cell proliferation, other mechanisms such as the modulation of cell death may be involved in the reduced tumor growth after combined treatment with gemcitabine and UAMC-2526. Apoptosis was not affected in Panc02 tumors by both compounds, yet UAMC-2526 inhibited tumor necrosis. Recent evidence indicates that autophagy is able to both inhibit and facilitate (regulated) necrosis, depending on the conditions (54). Given that tumor growth may result from rounds of disordered necrotic cell death, rather than from a process that is dictated solely by cell growth (35), it cannot be excluded that inhibition of necrosis by UAMC-2526 contributes to impaired Panc02 tumor growth.

In summary, UAMC-2526 potentiates the therapeutic effects of gemcitabine and significantly improves gemcitabine-mediated Panc02 tumor growth inhibition in mice.

## Data Availability Statement

The original contributions presented in the study are included in the article. Further inquiries can be directed to the corresponding author.

## Ethics Statement

The study was conducted according to the guidelines of the Declaration of Helsinki, and approved by the Ethics Committee of the University of Antwerp (file number 2017-68).

## Author Contributions

FST, CV, SS, PVDV, and WM contributed to the conceptualization of the experiments. FST, CV, MT, SDB, and IP conducted the experiments FST and GRYDM performed statistical analyses FST drafted the manuscript WM, MB, GRYDM, SS, and PVDV delivered acquisition of financial support for the project leading to this publication and critically reviewed and edited the manuscript. All authors have read and agreed to the published version of the manuscript.

## Funding

This research was funded by the University of Antwerp (IOF SBO, grant number 34927, BOF SEP 41477 and iBOF project ID 21-053) and the Fund for Scientific Research-Flanders (grant G.0412.16N). The FEI Tecnai transmission electron microscope was purchased with support of the Hercules Foundation.

## Conflict of Interest

The authors declare that the research was conducted in the absence of any commercial or financial relationships that could be construed as a potential conflict of interest.

## Publisher’s Note

All claims expressed in this article are solely those of the authors and do not necessarily represent those of their affiliated organizations, or those of the publisher, the editors and the reviewers. Any product that may be evaluated in this article, or claim that may be made by its manufacturer, is not guaranteed or endorsed by the publisher.
